# VEP Responses to Op-Art Stimuli

**DOI:** 10.1371/journal.pone.0139400

**Published:** 2015-09-30

**Authors:** Louise O’Hare, Alasdair D. F. Clarke, Petra M. J. Pollux

**Affiliations:** 1 School of Psychology, University of Lincoln, Lincoln, United Kingdom; 2 School of Psychology, Univerisity of Aberdeen, Aberdeen, United Kingdom; State University of New York Downstate Medical Center, UNITED STATES

## Abstract

Several types of striped patterns have been reported to cause adverse sensations described as visual discomfort. Previous research using op-art-based stimuli has demonstrated that spurious eye movement signals can cause the experience of illusory motion, or shimmering effects, which might be perceived as uncomfortable. Whilst the shimmering effects are one cause of discomfort, another possible contributor to discomfort is excessive neural responses: As striped patterns do not have the statistical redundancy typical of natural images, they are perhaps unable to be encoded efficiently. If this is the case, then this should be seen in the amplitude of the EEG response. This study found that stimuli that were judged to be most comfortable were also those with the lowest EEG amplitude. This provides some support for the idea that excessive neural responses might also contribute to discomfort judgements in normal populations, in stimuli controlled for perceived contrast.

## Introduction

Visual discomfort is an umbrella term describing the adverse effects encountered on viewing certain visual stimuli, such as striped patterns (e.g. [1]), filtered noise patterns [2][3][4] and text [5]. Reported sensations include headache, eyestrain, and perception of illusory shapes and colours [1]. Discomfort encompasses many different sensations from multiple possible sources [6]. For example, one source of discomfort is from accommodation-vergence conflict in stereoscopic images [7]. Another suggested source of discomfort is from eye movements, for example convergence insufficiency, particularly in the case of text [8].

The sensation of shimmering is included under the term ‘visual discomfort’, and there are several illusions that cause reports of shimmering in observers. Examples include MacKay’s radial lines [9], as well as more recently the rotating-tilted-lines illusion [10][11] and the accordion grating illusion [12], [13]. These illusions are of particular significance as those with developmental dyslexia are less susceptible to such motion illusions, which could make these tests an indicator of a deficit with gene DCDC2, a gene associated with the magnocellular pathway [14]. Some op-art artists create works that evoke shimmering illusions, such as work by Debbie Ayles, and Bridget Riley [2]. Op-art has been studied by researchers in order to investigate the causes of these illusory effects [15]. In particular, Leviant’s illusion ‘Enigma’, has been the centre of scientific debate. There are two types of illusory movement arising from this artwork: circular motion from the rings and shimmering illusions from the radial lines [16]. It has been argued that the shimmering illusion is due to fluctuations in accommodation, the focussing response of the eye [17]. It was also suggested that very small eye movements called microsaccades have an important role to play in the perception of shimmering [18]. Empirical evidence has been found to support this claim in the case of the Enigma illusion [19], as well as other illusions of motion based on geometric patterns [20][21][22].

Microsaccades are a class of eye movements typically found during attempted fixation. One possible purpose for these fixational eye movements is to prevent the visual stimulus from fading (as a consequence of adaptive processes) [23][24][25], and to facilitate binocular fusion [26]. Others have suggested that these movements have no purpose [27]. Mircosaccades tend to occur at a rate of 1Hz [28]. However, approximately 50–150ms after presentation of a visual stimulus there is a period of suppression, in that the frequency of microsaccade occurrence is reduced. It is important to note microsaccades are not eliminated. This period is followed by an increase in frequency of occurrence (rebound) in the time 200–300ms after stimulus onset [29]. This pattern is called the microsaccade signature [30][31].

**Fig 1:**
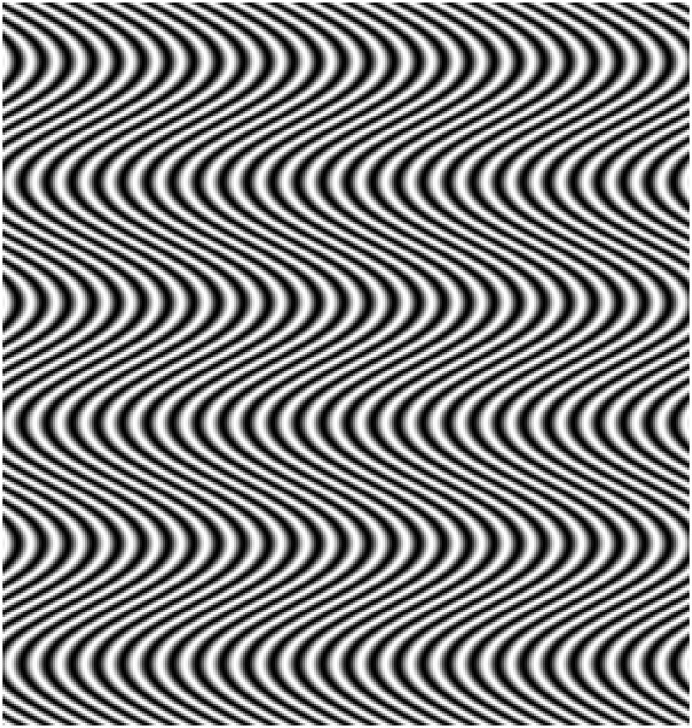
Example riloid stimulus.

The role of microsaccades in causing visual discomfort and shimmering effects in op-art has been investigated in a series of studies using stimuli based on the work of Bridget Riley, [32][33][16], see Fig 1 for an example. These stimuli are called ‘riloids’ and are defined by Eq (1). It is possible to modulate the spatial frequency of the underlying grating by varying (*λ*), and to manipulate the waviness of the curves by varying either the modulation amplitude (A) or wavelength (*μ*). Waviness of the curves (either varying amplitude (A) or by varying wavelength (*μ*)) was found to predict the magnitude of the illusory effects reported by observers. This is explained by the 2-dimensional visual-motion detection (2DMD) model [34], which uses an array of motion detectors and signal detection theory to analyse an image and determine the motion signals in the image that can be used to drive eye-movements. Further evidence for the role of microsaccades in the illusion was obtained by using flash stimuli or pinhole displays to reduce or negate the effects of these eye-movements. When the image was stabilised on the retina (using a flash) or the effects of accommodation reduced (by using a pinhole), the strength of the shimmering illusion also diminished [32]. As well as illusions based on the Enigma illusion, and the work of Bridget Riley, other illusions of motion are thought to rely on similar processes: eye movements and local motion integration processes are thought to be responsible for the illusory movement produced by the Ōuchi-Spillmann illusion [35]. This suggests that eye-movements, and eye-movements signals from these patterns, play a role in subjective effects resulting from these particular stimuli.

While the above evidence suggests a causal role of eye movements in shimmering illusions, there are indications that other factors that play a role in the perception of discomfort. For example, the 2DMD model cannot account for the shimmering reported while viewing straight lines [1]. Also, there are other subjective effects included in the term discomfort that are not readily explained by the 2DMD eye movement explanation [36]. Another explanation for discomfort is due to excessive neural responses [3], which might arise as a result of inefficient processing. Investigations into discomfort in general (not restricted to purely shimmering) arising from abstract images has shown that typically, stimuli judged as uncomfortable are ‘unnatural’—their spatial properties deviate from those typical of the kinds of images seen in everyday viewing [2][3]. Natural images generally have a 1/f^*β*^ amplitude spectrum, where contrast amplitude falls with increasing spatial frequency with *β* approximately 1—in the range 0.8 to 1.2 (e.g. [37]). Thus one metric for defining unnaturalness is deviation from the 1/f amplitude spectrum—slope values further from 1, or non-linear amplitude spectra.

There is a large amount of literature suggesting that the visual system exploits the properties typical of natural images in order to process them efficiently e.g. [38]. One example of an efficient code is sparse coding. In a sparse code, most of the neurons will not respond strongly to a stimulus, whereas a few will convey most of the information by responding strongly. This limits the number of active units needed to transmit the information, thus conserving metabolic resources as far as possible. Evidence supporting this theory has come from the fit of cells to the properties of images, for example [39][40][41]. Sparse coding has been applied to account for the aesthetic appeal of both paintings e.g. [42][43][44] and buildings [45]. According to sparse coding, works of art occupy a sub-range of the variability seen in everyday images, and is therefore able to be processed even more efficiently than natural images, resulting in the artwork being considered ‘pleasing to the eye’ [42]. The converse of this argument would follow that unnatural images are difficult to process, thus cause discomfort [3][46]. Spatial frequency content has been demonstrated to have a role in discomfort arising from both striped patterns [1] and filtered noise patterns [2]. Additionally, spatial frequency filtering can account for distortions in other, non-shimmering illusions [47], and has been suggested as an explanation for the Oppel-Kundt illusion [48]. Zeki and colleagues demonstrated increased neural responses to the Enigma illusion with their PET study [49]. Subsequent evidence also supports the idea of a role of the cortex in the perception of the illusion [50] using both behavioural [51] and neurostimulation techniques [52]. Therefore, there is evidence for a contribution of neural responses to the shimmering illusions.

At this point it should be highlighted that these different accounts, eye movement signals and inefficient coding, are not mutually exclusive, moreover, it is likely that they coexist. The different accounts could explain different aspects of visual discomfort, alternatively it is possible that microsaccades cause the neural responses. Although eye movements and neural responses cannot be completely separated experimentally, the two spatial parameters of riloids have slightly different predictions as to which discomfort mechanism they would predominantly influence. Stripe waviness (waviness *μ*) has already been predicted [34] and demonstrated [32][33][16] to affect illusion strength from spurious eye movement signals. However, previous work on discomfort in filtered noise would have predicted the spatial frequency (*λ*) of the underlying sine wave of the riloid pattern to have an effect on discomfort, possibly through inefficient coding [3], [46]. If it is the case that excessive responses are the cause of discomfort in some images, then this should be measurable from electroencephalogram (EEG) responses.

Visual evoked potentials (VEP) are early evoked components over the occipital areas of the brain occurring in response to visual stimuli. One typical component is the P100: a positive peak at a latency of approximately 100ms. This component is thought to be associated with early visual processing, which is the stage of processing where the theory of sparse-coding applies [53]. EEG results show increased amplitude VEP [54][55] and differences in latency [56] to those spatial frequencies to which humans are most sensitive. However, it is important to consider potential confounding variables. It has been demonstrated behaviourally that the visual system is differentially sensitive to spatial frequency content, leading to differences in perceived contrast e.g. [57]. The differences in perceived contrast follow a tuning function for spatial frequency at low levels of overall contrast (i.e., near detection threshold), called the contrast sensitivity function e.g. [57]. This tuning function is less pronounced when the overall contrast is higher (i.e., clearly above detection levels). However, although the differences are more equalised at higher physical contrasts, the tuning function is not completely flat even at high contrasts [58], meaning that some spatial frequencies will simply appear to have higher contrast than others, even though physically they are the same. As contrast itself affects discomfort judgements of striped patterns [1], but effects of discomfort remain when using stimuli of equal perceived contrast [4], it is important to control for the effects of perceived contrast differences. Perceived contrast is also influenced by eye movements: Eye movements enhance the perception of high spatial frequency content of a scene [59], making the high spatial frequency content appear higher contrast than it physically is. Due to these potentially confounding issues, it is important to control for the effects of perceived contrast. Therefore, whether EEG responses match visual discomfort judgements in normal populations, once the effects of the contrast sensitivity function are accounted for, remains unclear.

One difficulty with this line of argument is that microsaccades themselves are also influenced by the spatial frequency content of the scene [60], therefore the two explanations appear difficult to discriminate between in the case of spatial frequency contributions. However, the sparse coding literature is mostly restricted to early visual processing, for example V1 [53]. Due to the spatial dispersion of EEG responses, it is not possible to locate the source location of the recorded activity precisely. However, P100 is a relatively early visual response, occurring approximately 100ms post-stimulus, when the fewest microsaccades are expected to occur. It must be emphasised that the influence of microsaccades cannot be separated from the ratings of discomfort from the observers, however, it would be expected that their influence on the P100 would be minimal.

In summary, the current study was designed to investigate whether there are greater VEP responses from op-art-based images judged as more uncomfortable compared to those that are considered more comforable. If there is any evidence for the contribution of inefficient coding in discomfort judgements, then spatial frequency manipulations will affect discomfort judgements and VEP response magnitude, when stimuli are matched for differences in perceived contrast.

## Materials and Methods

### Observers

Twenty young observers with corrected-to-normal vision took part in the study. None reported migraine or epilepsy. All experiments were in accordance with the Code of Ethics of the World Medical Association, and approved by the University of Lincoln ethics committee. Written informed consent was obtained from all participants, and participants were reimbursed for their time.

### Apparatus

EEG was recorded with 2048Hz sampling rate with a 64 channel Active Two system (BioSemi, Amsterdam), with 5 additional electrodes (two on the mastoids, two on the outer canthi of the eyes, and the final one infraorbital on the right eye). During recording channels were referenced to the common mode sense electrode (see http://www.biosemi.com/faq/cms&drl.htm for more details). Incoming recordings were low pass filtered at 100Hz, and high pass filtered at 0.16Hz to remove artefacts. Experiments were conducted in a darkened, electrically insulated room. Head movements were not restricted, but participants were asked to remain as still as possible, and to focus on the fixation cross.

Stimuli were presented using a Dell Optiplex 780, running Windows XP and a 22 inch Illyama HM204DTA Vision Master Pro 514 Diamondtron U3-CRT monitor, calibrated with a LS100 Mintola photometer. Screen resolution was 1024 x 786 pixels. All stimuli were generated and presented using MATLAB and the Psychtoolbox [61][62][63].

### Stimuli

Stimuli consisted of nine riloid patterns, based on the work of Zanker and colleagues [32][33][16], see Eq (1):

$$\begin{array}{c} I\left(x,y\right)=0.5(1+\sin2\pi \frac{x-\phi (y)}{\lambda }) \end{array}$$ (1)

Where:

$$\begin{array}{c} \phi \left(y\right)=A\sin2\pi \frac{y}{\mu (y)} \end{array}$$ (2)

Where: I(x,y) defines luminance as a function of the horizontal (x) and vertical position (y), resulting in a sine wave of frequency *λ*, which varied between approximately 0.5, 3, and 9 cycles/degree, with phase modulated as *ϕ* (y), amplitude A was 0.94°, and wavelength (*μ*) was straight (infinity approximated by 10000000000 pixels), 100, and 400 pixels (inf°, 2.93° and 11.74°). Stimuli were presented in a Gaussian-edged window subtending a visual angle central region 5.94° and *σ* of 0.74°. Patterns reversed at a rate of 1Hz, changing polarity every 500ms. Stimuli were presented centrally for 10s duration.

### Procedure

Prior to the main experiment, observers were asked to match stimuli for perceived contrast using a self-adjustment procedure. Details of this task can be seen in S1 Fig. Each individual was presented with their own perceptually matched stimuli based on their settings from the contrast matching task. There were 9 stimuli, 3 levels of waviness (*μ*) x 3 levels of spatial frequency (*λ*), flickered at 1Hz. Each stimulus was presented once per block in randomised order, and there were seven blocks, with each stimulus presented once, resulting in 20 x 7 = 140 reversals of the 1Hz flicker (one complete cycle consists of 2 reversals per second). Each stimulus was presented centrally for 10s. A red fixation cross was presented throughout, and observers were asked to keep as still as possible, fixating the cross. There was varying onset time for each trial to prevent EEG locking to the start of the presentation [64]. When the stimulus disappeared, the observer was asked to enter their rating responses using the computer keyboard. Participants could use any value between 0 and 99 to enter their response. Participants were not instructed how much of the scale to use, nor were they given any training in order not to bias their responses.

### Analysis

Results were analysed using BrainVision Analyser and MATLAB. Behavioural responses were subjected to a repeated-measures 2-way ANOVA, with spatial frequency (*λ*) and waviness (*μ*) as factors, each with three levels. EEG responses were resampled from an original sampling rate of 2048Hz to 256Hz, for ease of analysis, and band-pass filtered between 0.1 and 70Hz to remove gross artefacts [65] and notch filtered at 50Hz to remove possible line noise. Channels were re-referenced to the average of all channels. Each 10s segment was divided into 500ms epochs based on stimulus presentation time, and a 50ms baseline before stimulus onset was subtracted. Artefact correction [66] was used to remove eye movements from the data, using the right canthus and FP1 as a reference. A threshold of +/-100waviness *μ*V was used as criterion for automatic rejection of artefacts. In previous research, components of interest have included P1, N1, and P2 for describing the early response to spatial frequency tuned stimuli [54]. In the current study, a component with a peak amplitude in the range 90–110ms was defined as the component of interest, following previous literature e.g. [67], [65], which possibly corresponds to P100, and will be referred to as such. Greenhouse Geisser corrections were used where assumptions of sphericity were violated to adjust the degrees of freedom. Repeated-measures post-hoc t-tests were used to explore any significant findings. Bonferroni correction resulted in an alpha level of 0.008 (0.05/6).

## Results

### Discomfort Judgements

**Fig 2:**
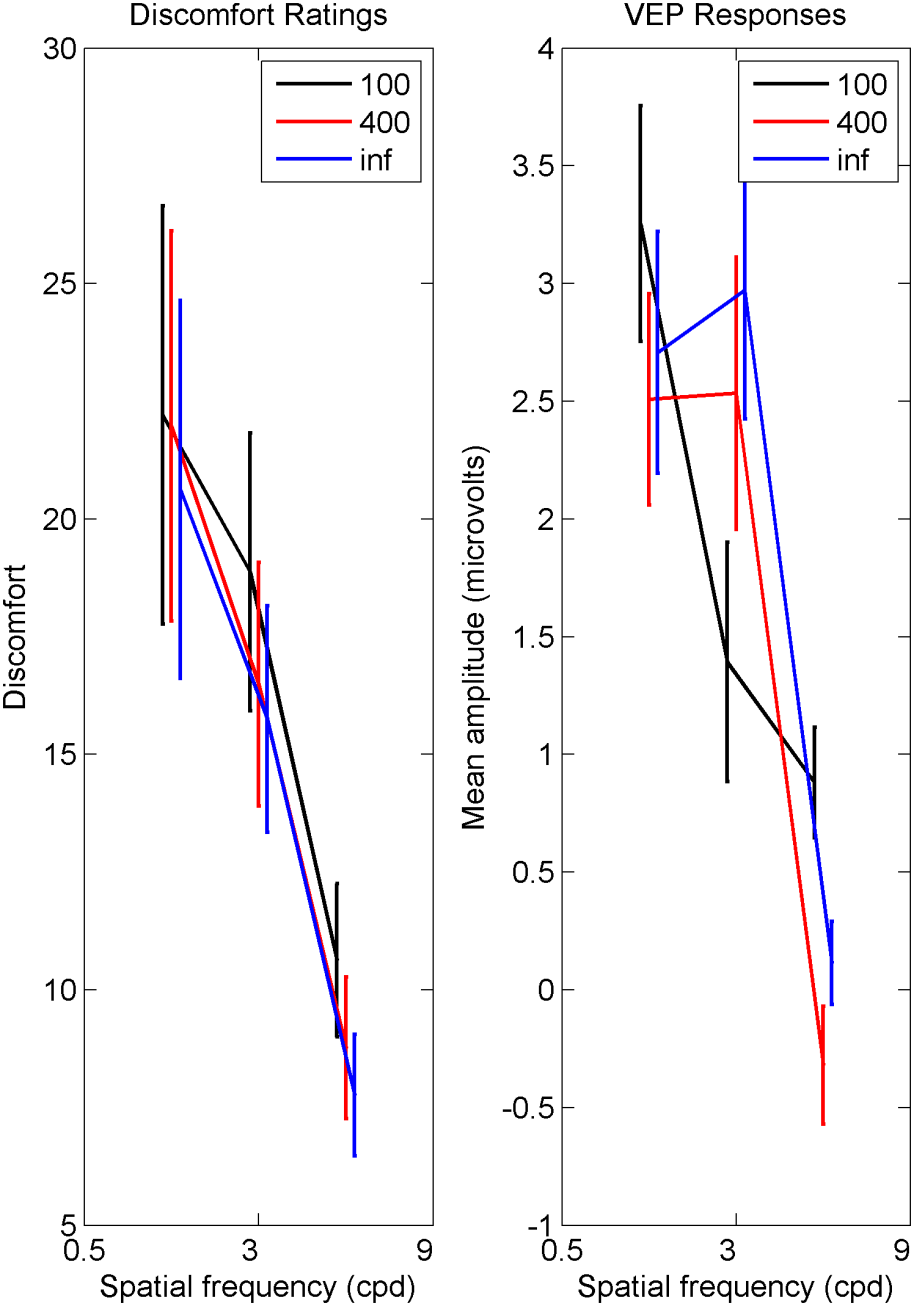
Discomfort judgements (left) and VEP amplitude (right). Left Mean discomfort judgements against spatial frequency (*λ*) for three levels of line waviness (*μ*). *μ* = 100 are the waviest, *μ* = inf are the straight lines. Error bars are one standard error. Right Mean VEP amplitude against spatial frequency (*λ*), for three levels of waviness (*μ*). *μ* = 100 are the waviest, waviness *μ* = inf are the straight lines. Error bars are one standard error.

Behavioural results are plotted on the left-hand side of Fig 2, which shows spatial frequency tuning for discomfort judgements. There is a main effect of spatial frequency (*λ*), and a main effect of waviness (*μ*), but no significant interaction between waviness (*μ*) and spatial frequency (*λ*). Post-hoc tests showed the tuning effect of spatial frequency to be driven by the high spatial frequency stimuli: Averaging over waviness (*μ*), the effect of spatial frequency was driven by the high spatial frequency stimuli being judged as less uncomfortable than the midrange spatial frequency stimuli, and the low spatial frequency stimuli. There is no significant difference in discomfort between the low and midrange spatial frequency stimuli when averaged over waviness. Post-hoc tests to explore the significance of waviness (*μ*) showed increased discomfort judgements for the waviest lines (*μ* = 100) compared to the straight lines (*μ* = inf) only. The difference between the two wavy lines (*μ* = 100 and *μ* = 400) was not significant, and the straight lines and the middling wavy lines (*μ* = inf and *μ* = 400) was also not significant. Overall, the highest spatial frequencies were less uncomfortable than both the other two spatial frequencies, and the waviest lines were more uncomfortable than the straight lines only. Statistics can be seen displayed in Table 1.

**Table 1:** Statistical Results.

**Discomfort Judgements**				
3 x 3 Mixed ANOVA	*F*	*df*	*p*	*η* ^2^
Spatial frequency (*λ*)	8.75	1.7,31.7	< 0.05	0.315
Waviness (*μ*)	8.40	2,38	< 0.05	0.307
Interaction (*λ* and *μ*)	1.02	4,76	NS	0.051
Post-hoc repeated measures t-tests	*t*	*df*	*p*	*r*
High vs low *λ*	3.56	19	< 0.008	0.589
High vs mid *λ*	4.95	19	< 0.008	0.709
Mid vs low *λ*	1.39	19	NS	0.282
100 vs 400 *μ*	2.50	19	NS	0.498
100 vs inf *μ*	3.43	19	< 0.008	0.618
400 vs inf *μ*	2.05	19	NS	0.426
**VEP Responses**				
3 x 3 Mixed ANOVA	*F*	*df*	*p*	*η* ^2^
Spatial frequency (*λ*)	18.90	2,38	< 0.05	0.499
Waviness (*μ*)	1.31	2,38	NS	0.065
Interaction (*λ* and *μ*)	8.73	4,76	< 0.05	0.315
Post-hoc repeated measures t-tests	*t*	*df*	*p*	*r*
High vs low *λ*	5.83	19	< 0.008	0.801
High vs mid *λ*	3.98	19	< 0.008	0.674
Mid vs low *λ*	1.46	19	NS	0.318
100 vs 400 *μ*	1.46	19	NS	0.318
100 vs inf *μ*	-0.42	19	NS	0.096
400 vs inf *μ*	-1.63	19	NS	0.350
**Eye Movements**				
3 x 3 Mixed ANOVA	*F*	*df*	*p*	*η* ^2^
Spatial frequency (*λ*)	2.55	2,38	NS	0.118
Waviness (*μ*)	2.55	2,38	NS	0.118
Interaction (*λ* and *μ*)	2.90	2.6,49.2	NS	0.132

### Visual Evoked Potentials

**Fig 3:**
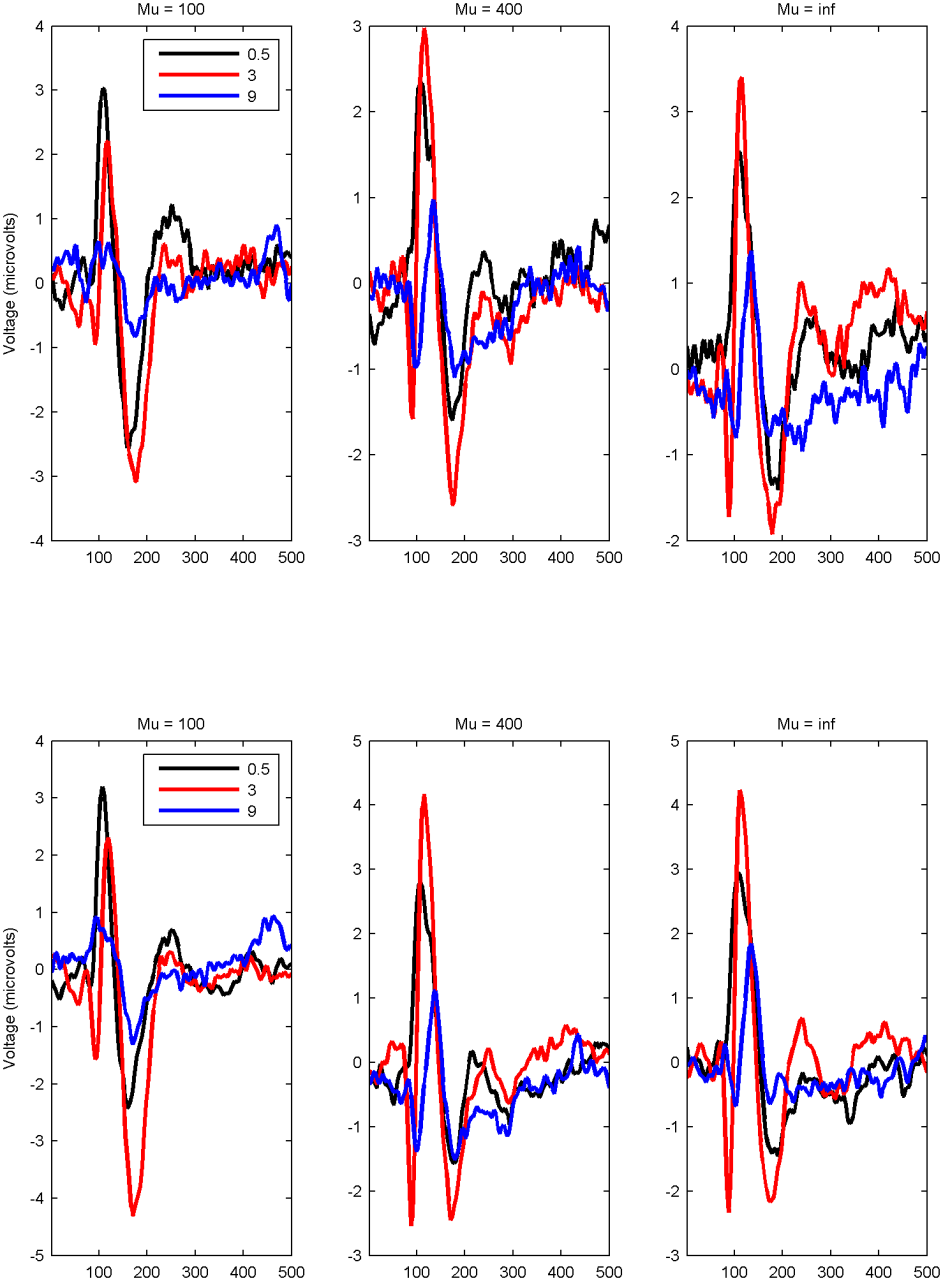
Time series of EEG. Mean EEG amplitude time series for three levels of spatial frequency (*λ*), for leftmost plot waviness (*μ*) = 100 (waviest lines) through to rightmost plot waviness (*μ*) = inf (straight lines). Top row = O1, bottom row O2.

The grand average EEG response for the electrodes O1 and O2 are plotted in Fig 3. This shows the EEG response averaged over valid trials, and then averaged over observers. As O1 and O2 were similar, results for O1 are reported. Fig 3 shows that signals returned to baseline before the end of the 500 ms epoch. The peak amplitude P100 response for each observer was calculated as the maximum amplitude of the positive response within the time frame 90–110ms (calculated from the baseline). This is plotted on the right hand side of Fig 2. There was a significant main effect of spatial frequency (*λ*) on amplitude of the P100 component. There was no effect of waviness (*μ*). The interaction between waviness (*μ*) and spatial frequency (*λ*) was significant. Post-hoc tests showed a similar pattern to the discomfort judgements for spatial frequency. Averaged over waviness, responses to high spatial frequency stimuli were lower on average than responses to low spatial frequency stimuli, and midrange spatial frequency stimuli. There was no difference between low and midrange spatial frequency stimuli. Although the right hand side of Fig 2 shows a different pattern for the waviest lines (*μ* = 100) compared to the other two, there are no significant differences between the three levels of line waviness. Overall, the highest spatial frequency stripes have lower P100 amplitude compared to the lower spatial frequencies. Statistics can be seen in Table 1.

### Eye movements

**Fig 4:**
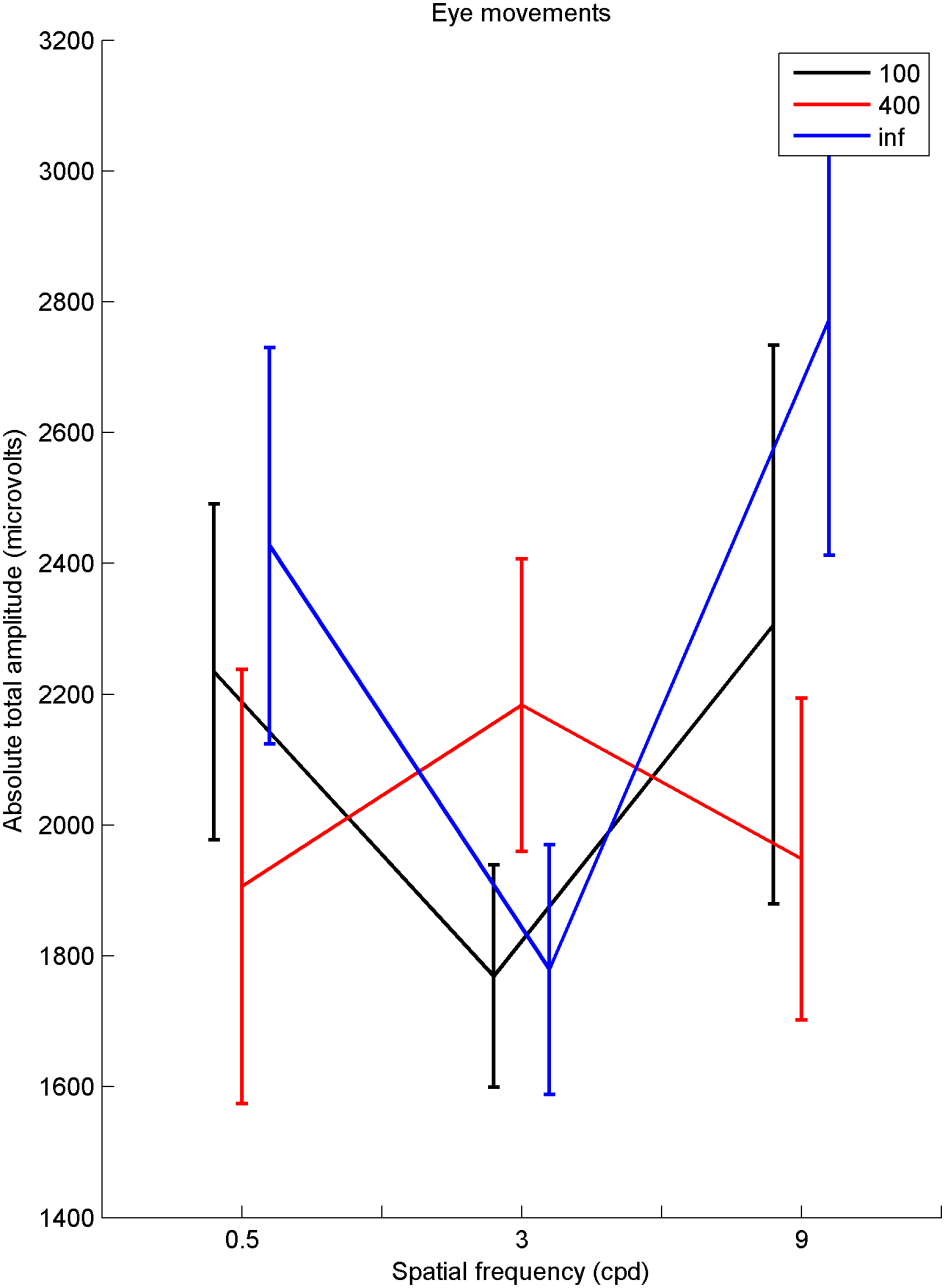
Eye movements. Mean amplitude for aggregate eye channel against spatial frequency (*λ*), for three levels of waviness (*μ*). (*μ*) = inf are straight lines. Error bars are one standard error.

Eye movements were analysed by taking the average of the three ocular (EOG) channels, and FP1, to form something similar to the ‘radial EOG’ of Keren et al., [68]. The aim was to see if there is any systematic variation in the magnitude of gross eye movements between stimuli. Pre-processing was minimal—the sampling rate of raw EEG was reduced to 256Hz and the EEG data was re-referenced to the average of all electrodes. The raw EEG was split into 500ms epochs. A measure was needed to see if there was any systematic variation between the nine stimuli for the overall amplitude of the eye movements. In order to characterise potential eye movements, the absolute value of the amplitude over each epoch was taken. The whole time series was integrated, to obtain one value for each repetition. The integral of each time series was then averaged over repetitions. Results of a 3 x 3 repeated measures ANOVA show no main effect of spatial frequency (*λ*), or waviness (*μ*), and no interaction on gross eye movements (see Table 1 for statistics). The results can be seen in Fig 4. It must be highlighted that this analysis will only demonstrate effects of gross eye movements, not any transient effects.

### Individual Differences

**Fig 5:**
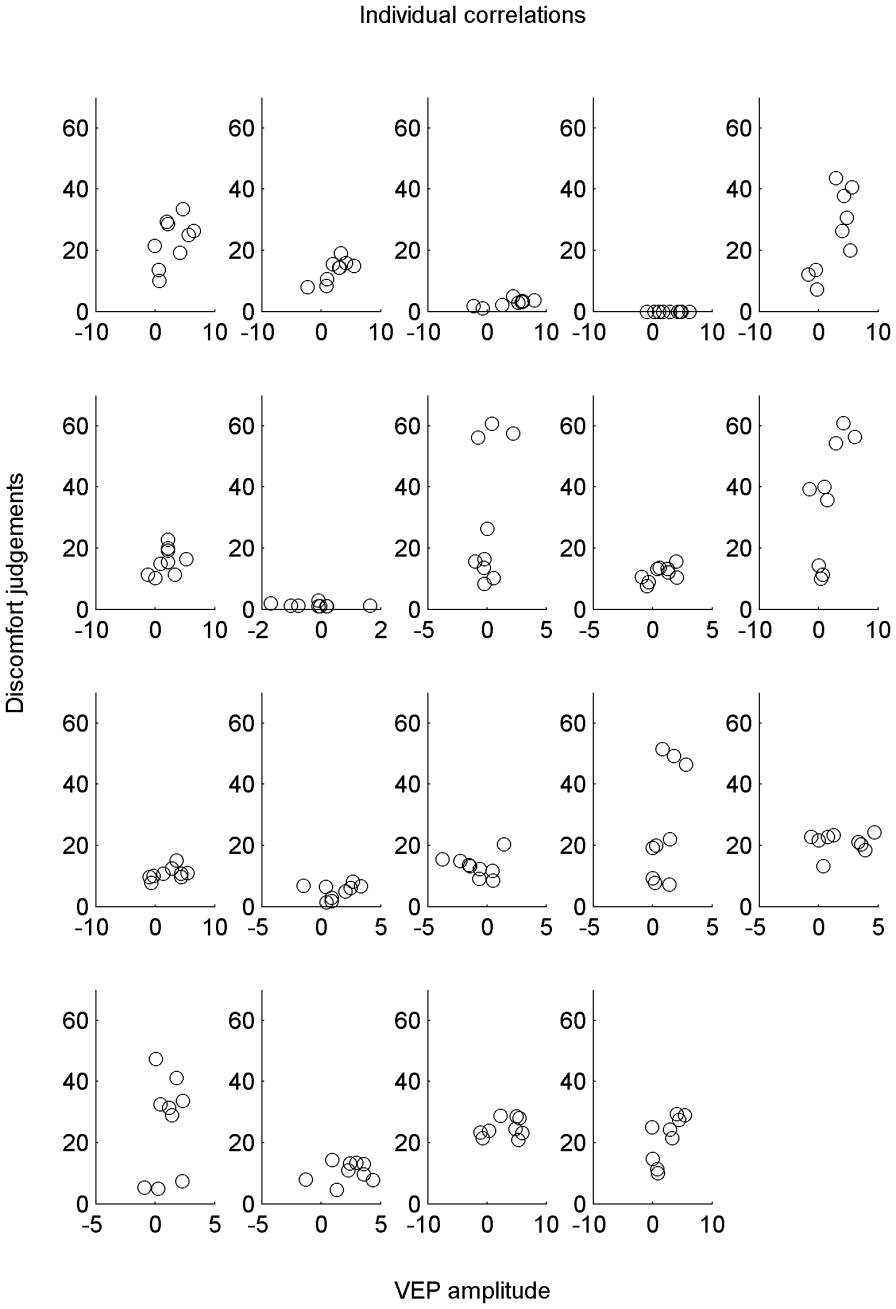
Individual correlations between discomfort judgements and VEP amplitude. Correlations between discomfort judgements and VEP amplitude for each of the nine stimuli (each stimulus represented by one point), plotted individually.

Fig 5 shows the amplitude of the EEG response against average subjective ratings for each individual observer. Each observer made a discomfort judgement at the end of each 10s presentation, and each presentation was repeated 7 times for each stimulus. The discomfort judgdments were averaged over each of the 7 repetitions of each 10s stimulus presentation period. The average P100 response for each 500ms epoch stimulus presentation within each of the 7 x 10s stimulation periods was calculated. Therefore each point on the plot represents the mean discomfort judgement and corresponding VEP amplitude for each of the nine stimuli. Although some individuals seem to show positive trends, there are too few data points (nine per observer) to reasonably investigate any relationship between them.

## Discussion

The aim of this study was to investigate the possibility of excessive neural responses, possibly arising from inefficient coding, contributing to visual discomfort in op-art-based stimuli. Neural response magnitude was defined as the peak amplitude of the P100 component of the VEP, discomfort was measured by observer ratings on a 0–99 scale, entered using a computer keyboard. Results showed that stimuli rated as less uncomfortable were also those with the lowest VEP amplitude, consistent with the predictions of excessive responses to uncomfortable stimuli. This result cannot be accounted for by contrast sensitivity alone, as the stimuli were first individually matched for perceived contrast. Additionally, the higher spatial frequencies were the ones with the highest physical contrast, thus in the opposite direction to be able to explain discomfort judgements.

In our stimuli we varied two parameters: spatial frequency (*λ*) and line waviness (*μ*). Participant ratings suggested that both parameters had an effect on perceived discomfort. The waviest lines were judged as more uncomfortable than either straight or middling wavy lines, consistent with the idea that effects of microsaccades, outlined by Zanker and colleagues [32][33][16], contribute to perceived discomfort judgements in these riloid stimuli. There was no effect of line waviness on P100 amplitude, as expected, as the P100 response would occur during the suppression period of microsaccades [28]. As the P100 differences are not immediately explicable by microsaccades, there is some support for the idea that excessive responses in the early visual areas contribute to discomfort judgements as well.

EOG signals were measured alongside the EEG, to ensure that large eye movements, which generally have a much stronger impact on the EEG signal than microsaccades, cannot explain the results. EOG signals were used in two ways: 1) to filter the EEG responses and 2) to examine any systematic relationship between the strength of the EEG and the strength of the EOG. There was no systematic effect of either spatial frequency (*λ*), or waviness (*μ*), on gross EOG amplitude averaged over the whole 500ms presentation. In the current experiment, observers were asked to fixate on the cross. When participants are informed their eyes are being tracked, they are able to maintain fixation [69]. There is also evidence that in highly trained observers, under certain conditions, microsaccades can be suppressed [23]. There are reports of some individuals being able to suppress their eye movements, and then perception of illusory movement is reduced [70]. Only simultaneous recording with a high performance eye tracker will fully exclude an explanation of the results on the basis of microsaccades. Observers may well have made fixational eye movements that could have contributed to their discomfort ratings. However, as microsaccades have only minor effects on the P100 response, and the suppression of microsaccades 100ms after stimulus onset [28], it is unlikely that microsaccades alone can account for the results.

There is some variation in the peak location for discomfort in this study compared to previous work e.g. [1], [4], [71]. This is possibly due to differences in the exact spatial and temportal characteristics of the stimuli used in the experiments, meaning it is not possible to make direct comparisons between studies for spatial frequency. As participants were not restricted by a chinrest, it is possible that there is some variation in the exact spatial frequencies presented to each participant. Thus the spatial frequencies used are approximate, e.g. as ‘high’, mid’ or low’ spatial frequency components. As there are only three stimuli in each manipulation (three levels of spatial frequency (*λ*), three levels of waviness (*μ*)), further work is needed to better characterise the tuning function.

While on a group level, there was a relationship between discomfort ratings and VEP amplitude, individual discomfort judgements were not predictable from EEG amplitude. It appears that some individuals might show positive trends, for example, the first, the bottom left, and the final individual (see Fig 5). Future studies should address this, particularly focussing on more measurements per participant. It is likely that the discomfort estimates will be noisy, as participants will have understood the scale differently. For example, differences in criterion might be accountable for the lack of direct mapping of discomfort judgements and EEG amplitude. Observers were given minimal instructions: They were reminded that there was no ‘correct’ answer, and that they could use as much of the scale as they felt necessary to report their perceived discomfort. No training was given on how to use the scale, in an attempt to avoid influencing observers’ judgements. It was understood that some observers might not feel any discomfort on viewing the stimuli, whilst others might find them much more challenging to look at. Additionally, EEG amplitude is not necessarily comparable across individuals, due to differences in electrical conductance, the underlying neuron formation, and electrode placement. Although removing the baseline reduces these effects, they will still be a level of noise in the EEG data. Due to difficulties in comparing absolute VEP amplitudes and subjective ratings across individuals, it is unsurprising that it is not possible to predict a particular individual’s discomfort judgements based on their EEG responses. However, from the group results, it is possible to say that some stimuli are, on average, challenging to look at for the normal population.

The entire experiment was conducted on individuals taken from the general population, in order to assess the natural variation in the population, without any initial screening. It might be possible to predict discomfort of different populations based on their VEP responses: The severity of reported discomfort varies between individuals [72]. It could be argued that screening the sample population for susceptibility to visual discomfort (for example, using the Pattern Glare Test [36]) might yield more extreme results, and therefore demonstrate a correlation between EEG amplitude and discomfort judgements. However, prescreening in this fashion might be considered circular, and therefore was not attempted. Discomfort judgements are typically more extreme for migraine populations (e.g. [73]). However, although there is also evidence that migraineurs tend to report more discomfort, those susceptible to visual discomfort actually represent a separate group [74], consisting of both migraineurs and headache-free individuals, therefore it would not be valid to consider migraine and visual discomfort groups as one and the same. The results of the current study show there is sufficient variation in the ratings, and EEG amplitudes to show results for the general population, where the effect will potentially be more subtle. This demonstrates an effect that is not restricted to clinical groups or other pre-screened subgroups.

The current study shows increased amplitude VEP responses for the same stimuli that are judged to be uncomfortable. This could be because uncomfortable stimuli result in non-sparse responses from non-natural spatial frequency characteristics. This is not in disagreement with previous research suggesting that shimmering illusions are due to microsaccades [18][32][33][16][19]. As Wade points out, op-art stimuli create a variety of perceptual effects, not just shimmering [15]. Mon-Williams and Wann also acknowledged there is a role of the brain in visual discomfort, in addition to shimmering illusions being most likely due to microsaccades [18]. As visual discomfort is a general term [6], and observers were not given additional explanation to their responses, it is possible that they would have considered both any shimmering and additional effects they experienced in their ratings. Future research would be needed to separate different aspects of discomfort and their causes, if, as suggested, they exist.

### Conclusion

VEP response amplitude corresponded to discomfort judgements in the case of high spatial frequencies, and this effect is not explicable by effects of perceived contrast. There is some support that excessive responses, possibly as a result of inefficient coding, can explain some aspect of discomfort from periodic stimuli in the general population. Line waviness (*μ*) also contributed to perceived discomfort. As line waviness did not affect magnitude of VEP responses, it appears that there are possibly two distinct contributors to perceived discomfort, eye movements as well as neural responses.

## Supporting Information

S1 Fig Mean settings for the contrast matching experiment. Contrast is shown against spatial frequency (*λ*) for three levels of waviness (*μ*). waviness (*μ*) = inf are the straight lines. Error bars show one standard error. All individuals were presented with their own contrast settings for the main experiment.
(TIF)

S1 Table Minimal data set. Anonymised data contained in Excel file available from figshare: http://dx.doi.org/10.6084/m9.figshare.1480915.
(XLSX)
