# Supplementary figures and images for: VEP Responses to Op-Art Stimuli
Source: PLoS One. 2015 Sep 30;10(9):e0139400. doi: 10.1371/journal.pone.0139400 (PMC4589386; doi:10.1371/journal.pone.0139400)

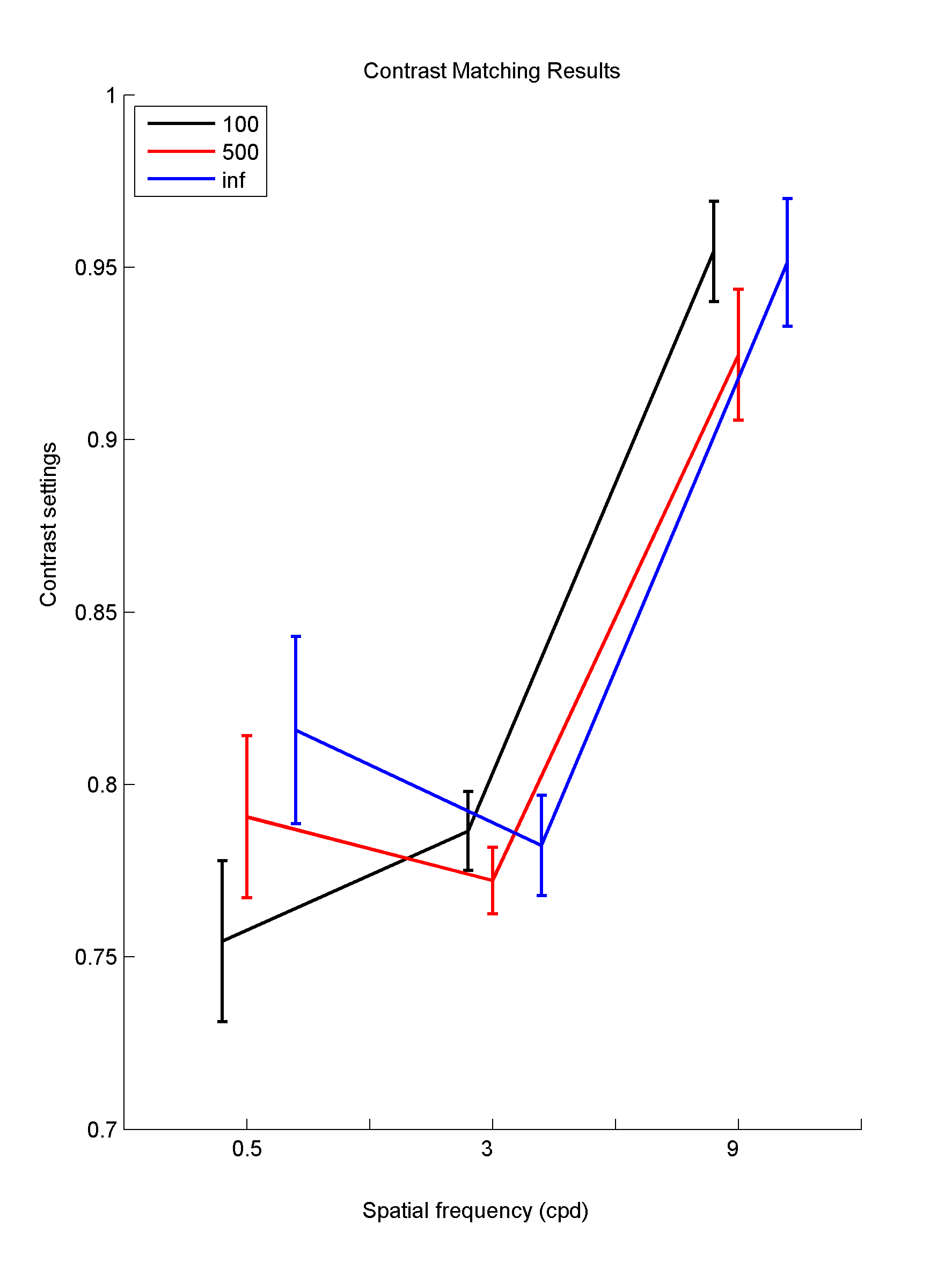

Supplement: S1 Fig — Contrast is shown against spatial frequency (λ) for three levels of waviness (μ). waviness (μ) = inf are the straight lines. Error bars show one standard error. All individuals were presented with their own contrast settings for the main experiment. (TIF) [file pone.0139400.s001.tif]
